# The performance of genome sequencing as a first-tier test for neurodevelopmental disorders

**DOI:** 10.1038/s41431-022-01185-9

**Published:** 2022-09-16

**Authors:** Bart P. G. H. van der Sanden, Gaby Schobers, Jordi Corominas Galbany, David A. Koolen, Margje Sinnema, Jeroen van Reeuwijk, Connie T. R. M. Stumpel, Tjitske Kleefstra, Bert B. A. de Vries, Martina Ruiterkamp-Versteeg, Nico Leijsten, Michael Kwint, Ronny Derks, Hilde Swinkels, Amber den Ouden, Rolph Pfundt, Tuula Rinne, Nicole de Leeuw, Alexander P. Stegmann, Servi J. Stevens, Arthur van den Wijngaard, Han G. Brunner, Helger G. Yntema, Christian Gilissen, Marcel R. Nelen, Lisenka E. L. M. Vissers

**Affiliations:** 1 Department of Human Genetics, Radboud University Medical Center, Nijmegen, The Netherlands; 2 Donders Institute for Brain, Cognition and Behaviour, Radboud University Medical Center, Nijmegen, The Netherlands; 3 Radboud Institute for Molecular Life Sciences, Radboud University Medical Center, Nijmegen, The Netherlands; 4 Department of Clinical Genetics, Maastricht University Medical Center, Maastricht, The Netherlands; 5 GROW School for Oncology and Developmental Biology, Maastricht University Medical Center, Maastricht, The Netherlands

**Keywords:** Neurodevelopmental disorders, DNA sequencing, Genetic techniques, Genomic analysis, Genetics research

## Abstract

Genome sequencing (GS) can identify novel diagnoses for patients who remain undiagnosed after routine diagnostic procedures. We tested whether GS is a better first-tier genetic diagnostic test than current standard of care (SOC) by assessing the technical and clinical validity of GS for patients with neurodevelopmental disorders (NDD). We performed both GS and exome sequencing in 150 consecutive NDD patient-parent trios. The primary outcome was diagnostic yield, calculated from disease-causing variants affecting exonic sequence of known NDD genes. GS (30%, *n* = 45) and SOC (28.7%, *n* = 43) had similar diagnostic yield. All 43 conclusive diagnoses obtained with SOC testing were also identified by GS. SOC, however, required integration of multiple test results to obtain these diagnoses. GS yielded two more conclusive diagnoses, and four more possible diagnoses than ES-based SOC (35 vs. 31). Interestingly, these six variants detected only by GS were copy number variants (CNVs). Our data demonstrate the technical and clinical validity of GS to serve as routine first-tier genetic test for patients with NDD. Although the additional diagnostic yield from GS is limited, GS comprehensively identified all variants in a single experiment, suggesting that GS constitutes a more efficient genetic diagnostic workflow.

## Introduction

Despite the diagnostic improvement of routine exome sequencing (ES) in clinical diagnostic laboratories, only about a third of the patients with neurodevelopmental disorders (NDD) of presumed genetic origin receive a definitive diagnosis [1, 2]. This diagnostic uncertainty can have considerable impact on patients, parents and families [3], which can be extenuated by providing more definitive molecular diagnoses.

A major advantage of genome sequencing (GS) over ES is the uniform sequence coverage across the entire genome [4] due to the elimination of enrichment artefacts [5], resulting in improved variant calling, especially for structural variation (SV) and short tandem repeats (STR) [6, 7]. This accurate and comprehensive analysis of all different variant types has great potential for further increasing the diagnostic yield. In rare disease patients in whom standard of care (SOC) testing failed to provide a molecular diagnosis, GS has been shown multiple times to reveal pathogenic variants leading to genetic diagnoses that were not identified at time of routine diagnostic work-up [8–14]. However, many of these studies were conducted in highly selected patient cohorts, making it difficult to draw firm conclusions on the potential of increased diagnostic yields when GS is employed in a prospective routine care setting [8–10]. While other studies have concluded that GS is technically suitable as first genetic test based on randomized control trials with standard of care [11–16], those studies have not allowed for a systematic comparison at the individual patient level for (type of) diagnoses gained or missed. As such, a prospective parallel study where patients receive both SOC and GS to determine the concordance between these two pathways and to address the question whether GS has added value over current SOC, has, to our knowledge, not yet been performed.

Here, we tested the hypothesis whether GS provides a higher diagnostic yield for patients with NDD when compared to current ES-based SOC. To this end, we prospectively studied 150 patient-parent trios with NDD, for whom we, in parallel to our (ES-based) SOC, performed GS. This not only allowed for a direct comparison of the clinical validity [17] of both genetic diagnostic pathways based on diagnostic yield, but also provided insights into the additive diagnoses made by either pathway, providing an unbiased evaluation of GS as first-tier genetic test for patients with NDD.

## Materials and methods

### Patient recruitment, selection and counseling

The departments of Human Genetics of the Radboudumc and MUMC+ are tertiary referral centers for patients with NDD in the Netherlands. A total of 150 consecutive index patients (*n* = 96 Radboudumc, *n* = 54 MUMC+) with neurodevelopmental delay of suspected genetic origin were included between October 1st 2018 and July 1st 2019. The only inclusion criterion was that the clinical geneticist requested a genetic diagnostic test to identify the molecular defect underlying the patient’s phenotype. Patients with a clinically recognizable syndrome (requiring genetic confirmation by a molecular genetic test) were not excluded from this study, making this cohort representative of the types of NDD patients seen in a tertiary referral center.

Board-certified clinical geneticists counseled all 150 patients and their parents for the ES-based SOC/GS procedures. All participants or their legal representatives gave written informed consent. This study was approved by the Medical Review Ethics Committee Arnhem-Nijmegen under 2011/188 and 2020-7142.

### Collection of family history and phenotypic information

Family history was negative for 145 cases, whereas for 5 patients, a recessive disorder or parental germline mosaicism was expected based on an affected sibling. Of note, if the genetic testing for both siblings started at the same time, making both sibs eligible for inclusion in this study, only one of the sibs was randomly selected and included. For all patients, phenotypic information was collected from the electronic health records and captured by standardized Human Phenotypic Ontology (HPO [18], release 2018-12-21) using PhenoTips (https://github.com/phenotips/phenotips, version 1.4.1).

### Standard of care procedures

**Fig. 1:**
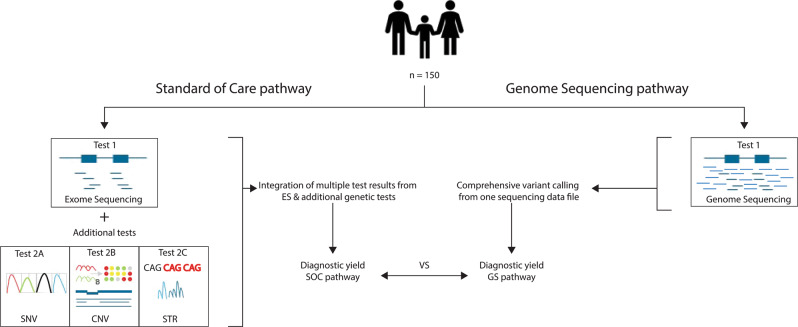
Flow scheme of the study of the performance of GS as first-tier test for neurodevelopmental disorders. All prospectively included patients and their parents received genome sequencing and the exome sequencing-based SOC work-up, supplemented with genomic microarray profiling and gene-by-gene testing upon request, allowing for a direct comparison of the performance of both pathways.

All patients received two diagnostic pathways in parallel, i.e., the (ES-based) SOC diagnostic pathway and the GS pathway (Fig. 1).

Requests for genetic diagnostic testing in the SOC pathway were left to the discretion of the clinical geneticist. The ES procedure was performed as described previously and included single nucleotide variant (SNV) and CNV analysis [19–21]. In brief, all DNA samples were sequenced with paired-end reads on an Illumina HiSeq 4000 or Illumina NextSeq 500 instrument in combination with Agilent version 5 enrichment kit to an anticipated median target coverage of 100-fold (Supplementary Table 1). ES could be supplemented with additional genetic tests, such as genomic microarray-based CNV profiling [22] (irrespective of CNV calling from ES), or targeted single gene-based testing for variants known to escape detection by ES (e.g., methylation assays for Prader Willi (OMIM 176270) or Angelman syndrome (OMIM 105830)), mitochondrial DNA testing, Sanger sequencing of individual genes not well-covered in ES, or repeat expansion analysis. Genetics tests performed prior to inclusion in this study were also considered part of the SOC pathway. Independent of the underlying genetic assay, genetic variants were prioritized and interpreted in the context of the patient’s phenotypic presentation, using routine procedures [2].

### GS procedure and bioinformatic analysis

GS was performed as described by the manufacturer (Illumina, San Diego, CA, USA). In brief, 1000 ng DNA, isolated from whole blood, was used for library preparation using the Illumina TruSeq DNA PCR-free protocol. Insert size was set at an average of 450 bp (Supplementary Fig. 1A) by shearing DNA using a Covaris E210. For efficient use of sequence capacity, barcoded indexing was included in the library preparation. Samples were equimolarly pooled, six samples on an Illumina S2 flowcell (*n* = 5 flowcells), 12 samples on a S4 flowcell (*n* = 3 flowcells), or 15 samples on a S4 flowcell (*n* = 27 flowcells), prior to sequencing using an Illumina NovaSeq6000^TM^ instrument to an anticipated genome-wide coverage of 50-fold (with a confirmed minimum coverage of 45-fold after mapping) (Supplementary Fig. 1B).

After sequencing, FASTQ files were processed through our in-house developed GS-pipeline. In short, reads were mapped to the human reference genome (GRCh37/hg19) using BWA (v.0.78) and the quality of the resulting BAM file was assessed using Qualimap (v.2.2.1) (Supplementary Table 2) [23]. Variant calling was performed per variant type using dedicated tools to optimize sensitivity, subsequently followed by variant annotation to facilitate variant interpretation.

For small variants, including SNVs and insertion/deletion events (indels), xAtlas (v.0.1) [24] was used and variants were annotated using an in-house developed pipeline. This variant annotation was performed using the Variant Effect Predictor (VEP V.91) and Gencode (V.34lift37) basic gene annotations. Frequency information was added from GnomAD (V.2.1.1) [25] and from an in-house database. In-house gene panel information, composed by multidisciplinary expert teams consisting of a clinical laboratory geneticist, a molecular geneticist, and a clinical specialist, was added for those genetic variants within a known disease gene. Additional annotations included CADD score (V.1.6), SpliceAI, OMIM and KEGG pathways. Runs of homozygosity were detected using PLINK (v.1.07) [26] with the following parameters: homozyg-window-het=3, homozyg-snp=50 and homozyg-kb=300.

SVs were called using Manta Structural Variant Caller (v.1.1.0; Illumina) [27], following a paired-end and split-read evidence approach for SVs identification. CNVs were called using Canvas Copy Number Variant Caller (v.1.40.0; Illumina) [28], which detects copy number gains or losses based on read depth. SVs and CNVs were annotated using an in-house developed pipeline based on ANNOVAR and Gencode (V.34lift37) basic gene annotations, and included frequency information from GnomAD (V.2.1) [25], 1000G (V.8) [29] and the GoNL SV database [30].

Short tandem repeats (STRs) were analyzed using ExpansionHunter (v.3.1.2) [31] with default settings, by extracting the relevant reads from a binary alignment file and realign them to the locus to genotype the STR site. As input loci, a standard diagnostic variant catalog containing 39 well-described disease-causing STR loci was used (Supplementary Table 3).

### GS variant filtering, prioritization and interpretation

We developed a strategy for the filtering, prioritization, and interpretation of genomic variants, based on variant type and mode of inheritance (Supplementary Tables 4, 5). Clinical interpretation of nuclear DNA was limited to exonic variants and splice site variants up to ±8 bp (Supplementary Fig. 2), thus including non-coding exons and UTRs but excluding deep intronic sequences.

### SNVs

We first optimized our SNV prioritization strategy based on quality control (QC) metrics by analyzing coverage, percentage variant allele frequency, and QC scores for ten child–parent trios unrelated to this project (Supplementary Fig. 3). Subsequently, we applied this strategy to our cohort of 150 patient-parent trios. We then extracted the coding SNVs (Supplementary Fig. 2) based on mode of inheritance, by filtering for de novo, homozygous, compound heterozygous and hemizygous variants (Supplementary Table 4). After filtering, the variants were prioritized (Supplementary Table 5) and evaluated in the clinical context of the patient, first focusing on prior disease-gene relationships for NDD, but also including variants in genes for which this was to be established (e.g., “candidate disease genes”). Variants considered clinically relevant were validated by routine Sanger sequencing.

### CNVs

Canvas and MANTA output files were filtered for de novo, homozygous and hemizygous CNVs. Cohort metrics were used to establish CNV filter strategies (Supplementary Fig. 4). CNVs were assessed in three categories 1) all variants in the NDD gene panel, 2) exon-containing variants in all disease-associated genes, or 3) all exon-containing variants >10 kb. In addition, autosomal recessive genes with de novo or heterozygous CNVs were checked for compound heterozygosity with SNVs. CNVs were validated with a 6.85 million probe CytoScan XON array (Applied Biosystems^TM^, Thermo Fisher Scientific).

### STRs

Clinical interpretation of STRs was limited to *AFF2* (Fragile XE syndrome; OMIM 309548), *ARX* (early infantile epileptic encephalopathy; OMIM 308350)*, CSTB* (progressive myoclonic epilepsy 1A; OMIM 254800), *EIF4A3* (Richeri-Costa-Pereira syndrome; OMIM 268305), *DMPK* (myotonic dystrophy; OMIM 160900) and *FMR1* (Fragile X syndrome; OMIM 300624) because of their NDD-associated phenotypes. Allele sizes for both repeats were extracted from the ExpansionHunter data for all patients. Genotypes were then compared to the locus-specific expansion thresholds (Supplementary Table 3). Alignments of the calls exceeding the thresholds were manually curated for sequencing coverage and read mapping quality (GitHub-Illumina/GraphAlignmentViewer). All remaining likely aberrant expansion alleles were confirmed using PCR followed by fragment length analysis and repeat-primed PCR.

### Mitochondrial DNA variants

Variants called on “chromosome M” (for mapping to mitochondrial DNA sequence) were extracted from the variant call files and subsequently compared to confirmed pathogenic mutations reported in MITOMAP (https://www.mitomap.org/foswiki/bin/view/MITOMAP/ConfirmedMutations).

### Definition and comparisons of diagnostic yield

The diagnostic outcome was determined at the individual patient level in both testing pathways independently (i.e., GS and SOC). The outcomes were based on guidelines from the Association for Clinical Genetic Science (ACGS), the Dutch Society of Clinical Genetic Laboratory Specialists (VKGL) [32], and European guidelines for constitutional cytogenomic analysis [33]:

1. No conclusive diagnosis: no obvious pathogenic variant(s) (of either variant type) were detected (in either genetic test) that could potentially explain disease.
2. Possible diagnosis obtained: a variant(s) of unknown significance was identified in a previously established disease gene that could explain the patient’s phenotype. Or alternatively, a pathogenic variant(s) in a candidate disease-gene(s) was identified with a potential relationship to (part of) the patient’s phenotype.
3. Conclusive diagnosis obtained: a (likely) pathogenic variant in a disease gene associated with the patient’s phenotype was detected, which explains the phenotype observed.

Data on diagnostic tests were independently analyzed by different teams and outcomes were *not* exchanged between the two pathways prior to reaching a conclusion in each of them separately. Diagnostic endpoints for each of the care pathways were compared and discussed *after* completion of the diagnostic quest in regular meetings with a multidisciplinary team including molecular geneticists, clinical genetic laboratory specialists, clinical geneticists, and pediatric neurologists.

## Results

### Cohort demographics

All 150 patients (101 males, 49 females) with complex NDD were included between October 2018 and July 2019 (Supplementary Fig. 5A). At the time of inclusion, the median age of patients in our cohort was 9 years and 6 months (range 1 year and 10 months–42 years and 7 months; Supplementary Table 1 and Supplementary Fig. 5B). Clinical phenotypes of the patients were captured using HPO terms, with a median number of 12 HPO terms per patient (range 2–44; Supplementary Table 6 and Supplementary Fig. 5C). To gain insight into the representativeness of this cohort versus patients referred to our tertiary referral center, we observed the broad phenotypic spectrum known to be associated with NDD (Supplementary Table 7) but recognized a depletion for epilepsy (*p* = 0.01, Fisher’s exact test with Bonferroni correction).

### SOC diagnostic yield

The SOC pathway consisted of ES for all 150 patients and their parents. In 105 patients, this was supplemented with additional genetic tests such as genomic microarray profiling (*n* = 63), *FMR1* expansion detection (*n* = 66), or other targeted gene-based testing (*n* = 25). On average, 2.3 genetic tests were performed per patient (range 1–8 tests; Supplementary Table 8).

A conclusive genetic diagnosis was obtained for 43 (28.7%) of 150 patients (Tables 1–3 and Supplementary Table 9). These diagnoses included 26 SNVs, 13 small InDels, 3 CNVs (ranging in size from 600 kb to 1.3 Mb), and one STR expansion in *FMR1* causing fragile X syndrome (OMIM 300624). All SNVs, InDels, and CNVs were detected using ES, while the *FMR1* repeat was detected through a gene-specific tandem repeat assay requested in addition to ES. In addition to the conclusive diagnoses, 31 patients (20.7%) received a possible genetic diagnosis, and for 76 patients (50.7%), no genetic cause was identified (Table 1 and Supplementary Table 9).

**Table 1:** Concordance of diagnoses obtained in the SOC and GS pathways.

Diagnosis by		GS			
		Yes	Possible	No	Total
SOC	Yes	43	0	0	43
	Possible	0	31	0	31
	No	2	4	70	76
	Total	45	35	70	150

**Table 2:** Conclusive SNV diagnoses.

Patient	Gender	Concordance	Gene	Genomic Mutation	cDNA	Protein	Inheritance
2	female	yes	*PACS1*	Chr11(GRCh37):g.65978677C>T	NM_018026.3:c.607C>T	p.(Arg203Trp)	de novo
12	male	yes	*SPEN*	Chr1(GRCh37):g.16257748_16257752del	NM_015001.2:c.5013_5017del	p.(Glu1671Aspfs*16)	de novo
30	male	yes	*TBR1*	Chr2(GRCh37):g.162273598T>A	NM_006593.3:c.677T>A	p.(Ile226Asn)	de novo
35	male	yes	*CDK13*	Chr7(GRCh37):g.39990724dup	NM_003718.4:c.484dup	p.(Ala162Glyfs*108)	de novo
			*TET3*	Chr2(GRCh37):g.74326613C>T	NM_001287491.2:c.3478C>T	p.(Arg1160Cys)	de novo
39	female	yes	*ANKRD11*	Chr16(GRCh37):g.89346232C>A	NM_001256182.1:c.6718G>T	p.(Glu2240*)	de novo
40	female	yes	*GATAD2B*	Chr1(GRCh37):g.153791300_153791301del	NM_020699.2:c.565_566del	p.(Gln190Alafs*34)	de novo
49	male	yes	*DOCK3*	Chr3(GRCh37):g.51127708_51127711dup	NM_004947.4:c.639_642dup	p.(Pro214Ilefs*7)	homozygous
55	male	yes	*ANKRD11*	Chr16(GRCh37):g.89347820dup	NM_001256182.1:c.5130dup	p.(Ser1711Ilefs*21)	de novo
57	male	yes	*SLC16A2*	ChrX(GRCh37):g.73641601del	NM_006517.3:c.351del	p.(Val118Cysfs*40)	de novo
63	female	yes	*MECP2*	ChrX(GRCh37):g.153296476del	NM_001110792.1:c.842del	p.(Gly281Alafs*20)	de novo
66	female	yes	*SHANK3*	Chr22(GRCh37):g.51159604C>T	NM_001080420.1:c.3391C>T	p.(Arg1131*)	de novo
67	male	yes	*CHD3*	Chr17(GRCh37):g.7803968G>A	NM_001005271.2:c.3074G>A	p.(Arg1025Gln)	de novo
71	male	yes	*TRIP12*	Chr2(GRCh37):g.230632449G>T	NM_001348328.1:c.6028C>A	p.(Pro2010Thr)	de novo
73	male	yes	*ATRX*	ChrX(GRCh37):g.76875938T>C	NM_000489.3:c.5197A>G	p.(Lys1733Glu)	hemizygous
74	male	yes	*ACTB*	Chr7(GRCh37):g.5568193G>A	NM_001101.4:c.521C>T	p.(Ala174Val)	de novo
79	male	yes	*BMP4*	Chr14(GRCh37):g.54418925G>A	NM_001202.3:c.16C>T	p.(?)	de novo
80	male	yes	*ANKRD11*	Chr16(GRCh37):g.89351038_89351039del	NM_001256182.1:c.1914_1915del	p.(His638Glnfs*24)	de novo
82	male	yes	*SHANK3*	Chr22(GRCh37):g.51159940dup	NM_001080420.1:c.3727dup	p.(Ala1243Glyfs*69)	de novo
84	female	yes	*MYCN*	Chr2(GRCh37):g.16082228C>A	NM_001293228.1:c.42C>A	p.(Cys14*)	de novo
86	male	yes	*WASF1*	Chr6(GRCh37):g.110422797G>A	NM_003931.2:c.1516C>T	p.(Arg506*)	de novo
88	male	yes	*CUL3*	Chr2(GRCh37):g.225371574C>G	NC_000002.12(NM_001257198.1):c.1047+1G>C	p.(?)	de novo
96	female	yes	*DDX3X*	ChrX(GRCh37):g.41205496dup	NM_001356.4:c.1330dup	p.(Thr444Asnfs*21)	de novo
97	male	yes	*SLC2A1*	Chr1(GRCh37):g.43395707C>A	NC_000001.11(NM_006516.2):c.517-1G>T	p.(?)	de novo
98	male	yes	*ABCD1*	ChrX(GRCh37):g.153002655C>A	NM_000033.3:c.1438C>A	p.(Pro480Thr)	hemizygous
102	female	yes	*DDX3X*	ChrX(GRCh37):g.41203054C>T	NM_001356.4:c.744C>T	p.(Gly248=)^a^	de novo
107	male	yes	*KDM5C*	ChrX(GRCh37):g.53223539C>T	NM_004187.3:c.3820G>A	p.(Glu1274Lys)	de novo
109	male	yes	*PPM1D*	Chr17(GRCh37):g.58734161dup	NM_003620.3:c.1219dup	p.(Cys407Leufs*27)	de novo
110	female	yes	*FOXP2*	Chr7(GRCh37):g.114292307C>T	NM_148898.3:c.1219C>T	p.(Arg407*)	de novo
113	male	yes	*KCNQ2*	Chr20(GRCh37):g.62076675G>A	NM_172107.2:c.430C>T	p.(Arg144Trp)	de novo
116	female	yes	*FBXO11*	Chr2(GRCh37):g.48132751G>A	NM_001190274.1:c.109C>T	p.(Gln37*)	de novo
120	male	yes	*ANKRD11*	Chr16(GRCh37):g.89349832T>A	NM_001256182.1:c.3118A>T	p.(Lys1040*)	de novo
121	female	yes	*DEAF1*	Chr11(GRCh37):g.686982C>T	NM_021008.3:c.680G>A	p.(Cys227Tyr)	de novo
124	male	yes	*KAT6A*	Chr8(GRCh37):g.41791983del	NM_006766.4:c.3756del	p.(Ser1253Leufs*41)	de novo
132	female	yes	*DDX3X*	ChrX(GRCh37):g.41206602C>T	NM_001356.4:c.1807C>T	p.(Arg603*)	de novo
133	male	yes	*GRIA3*	ChrX(GRCh37):g.122561802G>A	NM_000828.4:c.1888G>A	p.(Gly630Arg)	hemizygous
139	female	yes	*GNB1*	Chr1(GRCh37):g.1735941C>T	NM_002074.4:c.347G>A	p.(Gly116Asp)	de novo
140	male	yes	*NFIX*	Chr19(GRCh37):g.13184270del	NM_001271043.2:c.681del	p.(Val229Serfs*4)	de novo
144	male	yes	*FOXG1*	Chr14(GRCh37):g.29237130C>A	NM_005249.4:c.645C>A	p.(Phe215Leu)	de novo
146	male	yes	*KCNQ2*	Chr20(GRCh37):g.62044909G>A	NM_172107.2:c.1657C>T	p.(Arg553Trp)	de novo

^a^This synonymous variant causes alternative splicing and nonsense-mediated decay of the alternative out-of-frame transcript, which was found and validated in two prior in-house patients.

**Table 3:** Conclusive CNV and STR diagnoses.

Patient	Gender	Concordance	Gene	Genomic Mutation (GRCh37)	Size	Inheritance
33	female	GS+	*CHD2*	15q26.1(93494184-93499518)x1	5 kb	de novo
47	female	GS+	*AHDC1*	1p36.11(27833526-27869757)x1	36 kb	de novo
8	female	yes^a^	*CSNK2A1*	20p13(486287-1783151)x1	1.3 Mb	de novo
			*PDYN*	20p13(1963481-1995077)x3	32 kb	de novo
25	male	yes	*FMR1*	X:146993568_146993628CGG[94]	94 repeat units	anticipation
117	male	yes	Multiple	16p11.2(29591089-30199431)3x	0.6 Mb	paternal^b^
142	male	yes	Multiple	16p13.2(8165983-9074579)1x	0.9 Mb	de novo

^a^The pathogenic 20p13 deletion was found by both ES and GS. GS, however, revealed an additional 20p13 duplication.

^b^Recurrent 16p11.2 microduplication syndrome (OMIM 614671) with an estimated penetrance of 11.2% [42].

### GS diagnostic yield

Prospectively, and in parallel to the ES-based SOC pathway, all patients (and their parents) received GS, which was analyzed independently of the SOC. A total of 45 patients (30.0%) received a conclusive genetic diagnosis, consisting of 26 SNVs, 13 small InDels, 5 CNVs (ranging in size from 5 kb to 1.3 Mb), and one STR expansion in *FMR1* (Tables 1–3 and Supplementary Table 9). In addition, 35 patients (23.3%) received a possible diagnosis, and for 70 patients (46.7%), the genetic cause remained elusive (Table 1 and Supplementary Table 9).

### Comparison of GS to SOC

**Fig. 2:**
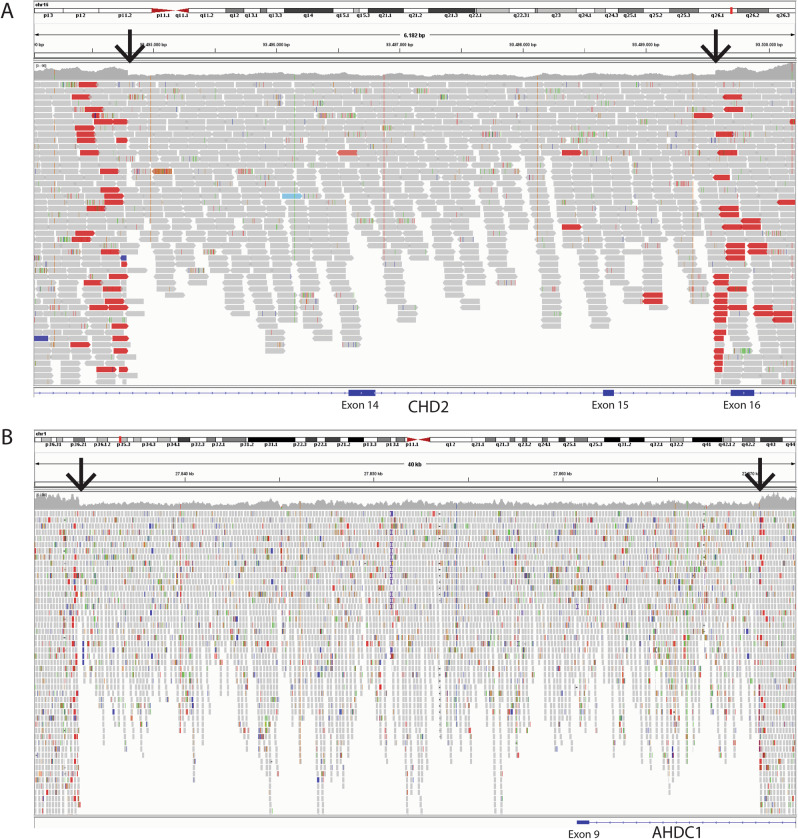
Genomic alignment of *CHD2* and *AHDC1* CNVs in genome sequencing data. Visualization of genomic alignment of GS data in the Integrative Genomics Viewer (IGV) software for two CNVs that were detected by GS only. **A** 5 kb deletion in *CHD2* at 15q26.1(93494184-93499518)x1 (patient 33), encompassing exon 14 and 15. **B** 36 kb deletion in *AHDC1* at 1p36.11(27833526-27869757)x1 (patient 47), including the (non-coding) exon 9 (3′UTR). CNVs are visible from multiple lines of evidence, including read depth (**A** + **B**; arrow) and read pairs with larger than expected insert size (**A**; reads in red).

To objectively determine the value of GS compared to ES-based SOC for NDD patients, we subsequently determined the concordance between the diagnosis obtained at individual patient level via the ES-based SOC and GS pathways (Table 1). In total, 45 (30%) unique patients received a conclusive diagnosis through either the SOC pathway or the GS pathway (Tables 2, 3 and Supplementary Table 9). For 43 of the 45 conclusive diagnoses, both pathways obtained the same diagnosis, while two patients received a conclusive diagnosis through the GS pathway only. There was no statistically significant difference between the diagnostic yield of the ES-based SOC pathway and the GS pathway (*n* = 43 vs. *n* = 45, respectively; *p* = 1.0 Fisher’s exact test). The genetic diagnoses that were not detected using the ES-based SOC pathway included a small 5 kb deletion covering exons 14 and 15 of *CHD2*, and a 36 kb deletion including the 3′UTR (non-coding) exon 9 of *AHDC1* (Fig. 2 and Table 3). Of note, for one additional patient, GS revealed a more complex rearrangement consisting of a deletion-duplication event, of which the 32 kb duplication was only detected by GS (Table 3).

Similar concordance between both pathways was obtained at the level of possible diagnoses: GS identified a possible diagnosis for 35 patients, of which 31 were also identified by the ES-based SOC pathway (Supplementary Table 10). Interestingly, all four possible diagnoses only detected by GS involved CNVs (Supplementary Table 11).

There were no variants of (potential) diagnostic relevance that were identified in the ES-based SOC pathway, but failed detection in GS.

## Discussion

For the last decade, molecular diagnosis of patients with rare genetic disorders has increasingly relied on the use of ES, resulting in ES being a first-line diagnostic test [1]. Using ES, on average 1 in 3 patients receives a genetic diagnosis [1, 2]. For patients in whom the genetic etiology remains elusive, it has been shown that GS can help to provide a diagnosis, as it allows for comprehensive variant calling of all types of variation without the technical biases and limitations observed in ES [4–6, 8–13]. From our prospective parallel study including 150 patient-parent trios, we conclude that GS does not result in a significantly higher diagnostic yield (30.0%) than an ES-based SOC pathway (28.7%). Whereas 1.3% additional conclusive diagnostic yield in GS is not statistically significant from SOC, we do show that even with variant interpretation limited to “low hanging fruit” (i.e., those affecting exonic sequence and previously reported (likely) pathogenic non-coding variants), GS revealed more diagnoses than current SOC. Of note, to reach statistical significance for a 1.3% difference in diagnostic yield, our study would have required a cohort size of 32,039 patients, hence it may be a matter of time to prove diagnostic increase in GS. Our observations agree with recent randomized controlled studies where the diagnostic yield was also not significantly different from SOC [14, 15], although these studies were not able to show the direct comparisons of diagnoses obtained.

The overall diagnostic yield in our cohort (30%) is also in line with recent meta-analyses, reporting conclusive diagnostic yields between 25% and 36% for routine NDD cohorts [1, 19]. The expectation might have been that the overall conclusive diagnostic yield of GS would be 40–50% when based on cumulative diagnostic yields of each individual diagnostic strategy alone [9]. A possible explanation for not observing the cumulative yield is likely the absence of stringent clinical preselection of our cohort, which allowed the inclusion of any patient with NDD for whom ES-based genetic diagnostic testing was requested. Consequently, patients with phenotypes featuring the entire spectrum of NDD from mild, to moderate, to severe intellectual disability (ID), and with or without additional congenital anomalies, were included. The previous cumulative estimate of GS was, however, based on severe ID [9]. Another explanation for the seemingly lower than initially expected GS diagnostic yield may be the limited scope of variant interpretation, i.e., only focusing on variants affecting coding sequence using an “exome-from-genome” approach. Whereas examples of non-coding disease-causing variants most certainly exist [8, 34–36], the contribution of such non-coding variants for NDD have so far been estimated to be rare [12, 37, 38]. In addition, interpretation and establishing pathogenicity of variants in non-coding space is not straightforward [39], and often involves labor-intensive and time-consuming (functional) follow-up experiments to determine putative clinical relevance [34, 38, 40]. Such experiments are mostly performed in a research setting, where time is less of an issue than in routine diagnostic settings.

In the GS data, we observed a 100% concordance for clinically relevant variants identified by ES-based SOC (i.e., GS detected all 43 conclusive diagnoses as well as all 31 possible diagnoses identified in ES-based SOC). The 84 variants underlying these 74 diagnoses included SNVs (67.9%), InDels (20.2%), CNVs (10.7%) and repeat expansions (1.2%). Interestingly, however, the two conclusive and four possible diagnoses only identified through GS, all represented CNVs, being a significant overrepresentation from expectation (*P* = 7.823e-06, Fisher’s exact test), suggesting that CNVs might have an important role in the missed diagnoses of rare NDDs. The CNVs were not identified in ES due to limitations in sensitivity and specificity of CNV variant calling in ES (*n* = 4) or due to the absence of targets in the ES enrichment procedure (*n* = 2). Our results thus emphasize the value of equal coverage of the entire human genome in GS for structural variant calling and breakpoint determination at base-pair resolution. It is therefore within reason to expect that improved structural variant calling, filtering and prioritization tools will increase the diagnostic yield of GS.

Our results show that GS comprehensively identified all variant types in a single laboratory experiment. The limited additional diagnostic yield of GS indicates that the ES-based SOC was already of high quality. However, for 70% of the patients, ES was supplemented with additional genetic assays. In order to replace the SOC pathway, GS must not only replace ES, but also most (if not all) genetic assays performed. Such a “one-test-approach” could benefit clinical laboratories where it is common that more than one test is being performed in NDD patients. A “one-test-approach” could also reduce the time to diagnosis for patients as these tests are not always performed in parallel, but in a stepwise (consecutive) fashion. Exceptions to this assumption are variants that cannot be currently captured by GS, such as methylation defects.

Besides the advantages of GS over the ES-based workup, performing GS is also associated with certain challenges that could possibly hamper the implementation of GS in routine diagnostic genetic testing. Two of these challenges are the data interpretation of large numbers of variants and the cost of sequencing. We show that the difficulty of interpreting GS data can be easily overcome by a tiered approach for interpretation where first variants in the “exome from genome”, CNVs and other “low hanging fruit” can be analyzed. When this does not yield a genetic diagnosis, the variants in non-coding space can be analyzed subsequently without having to perform novel sequencing. The cost of performing GS, although not assessed in detail in this study, are higher than ES, making wide-spread implementation of GS, as replacement for ES-based SOC, difficult despite the obvious advantages [41]. A detailed cost analysis that also factors in the possible cost reductions from assays that are no longer performed in a GS-first approach is therefore warranted. In addition, such analysis may provide insights into the tipping point under which circumstances GS can be implemented cost-neutrally. Alternatively, an elevation in diagnostic yield from GS due to increasing knowledge of pathogenic variants in non-coding space may also help justify the perceived increase in healthcare-associated costs.

In conclusion, our data demonstrate the technical and clinical validity of GS to serve as routine first-tier genetic test for patients with NDD. Despite the similar diagnostic yields observed between GS and SOC, GS successfully identified all clinically relevant variants in a single test. This contrasts with current routine testing which uses multiple tests to reach the same conclusions while potentially taking more time, suggesting that GS is a more efficient workflow. Whether this increased efficiency, in absence of a significantly increased diagnostic yield, can compensate for the expected increase in costs when considering wide-spread implementation of GS, remains to be determined.

## Supplementary information

Supplementary Methods, Figures and Tables

Supplementary Table 1

Supplementary Table 6

Supplementary Table 8

Supplementary Table 9

## Data Availability

The datasets used and/or analyzed during the current study are available from the corresponding author on reasonable request.
