# Supplementary material for: The performance of genome sequencing as a first-tier test for neurodevelopmental disorders
Source: Eur J Hum Genet. 2022 Sep 16;31(1):81–8. doi: 10.1038/s41431-022-01185-9 (PMC9822884; doi:10.1038/s41431-022-01185-9)
Supplement: Supplementary file 1 — Supplementary Methods, Figures and Tables [file 41431_2022_1185_MOESM1_ESM.docx]

Supplement to: **The performance of genome sequencing as first-tier test for neurodevelopmental disorders**

Bart P.G.H. van der Sanden^1,2^*, Gaby Schobers^1,2^*, Jordi Corominas Galbany^1,3^*, David A. Koolen^1,3^, Margje Sinnema^4^, Jeroen van Reeuwijk^1,2^, Connie T.R.M. Stumpel^4,5^, Tjitske Kleefstra^1,2^, Bert B.A. de Vries^1,2^, Martina Ruiterkamp-Versteeg^1^, Nico Leijsten^1^, Michael Kwint^1^, Ronny Derks^1^, Hilde Swinkels^1^, Amber den Ouden^1^, Rolph Pfundt^1^, Tuula Rinne^1^, Nicole de Leeuw^1,2^, Alexander P. Stegmann^4^, Servi J. Stevens^4^, Arthur van den Wijngaard^4^, Han G. Brunner^1,2,4,5^, Helger G. Yntema^1,2^, Christian Gilissen^1,3#^, Marcel R. Nelen^1#^, Lisenka E.L.M. Vissers^1,2#^

^1^Department of Human Genetics, Radboud university medical center, Nijmegen, The Netherlands

^2^Donders Institute for Brain, Cognition and Behaviour, Radboud university medical center, Nijmegen, The Netherlands

^3^Radboud Institute for Molecular Life Sciences, Radboud university medical center, Nijmegen, The Netherlands

^4^Department of Clinical Genetics, Maastricht University Medical Center, Maastricht, The Netherlands

^5^GROW School for Oncology and Developmental Biology, Maastricht University Medical Center, Maastricht, The Netherlands

*these authors contributed equally

# these authors jointly supervised the work

Corresponding author:

Lisenka E.L.M. Vissers, PhD

Radboudumc, Dept. of Human Genetics

PO Box 9101, 6500 HB

Nijmegen, the Netherlands

Email: [lisenka.vissers@radboudumc.nl](mailto:lisenka.vissers@radboudumc.nl)

Phone: +31 24 366 8163

**Supplementary Figures**


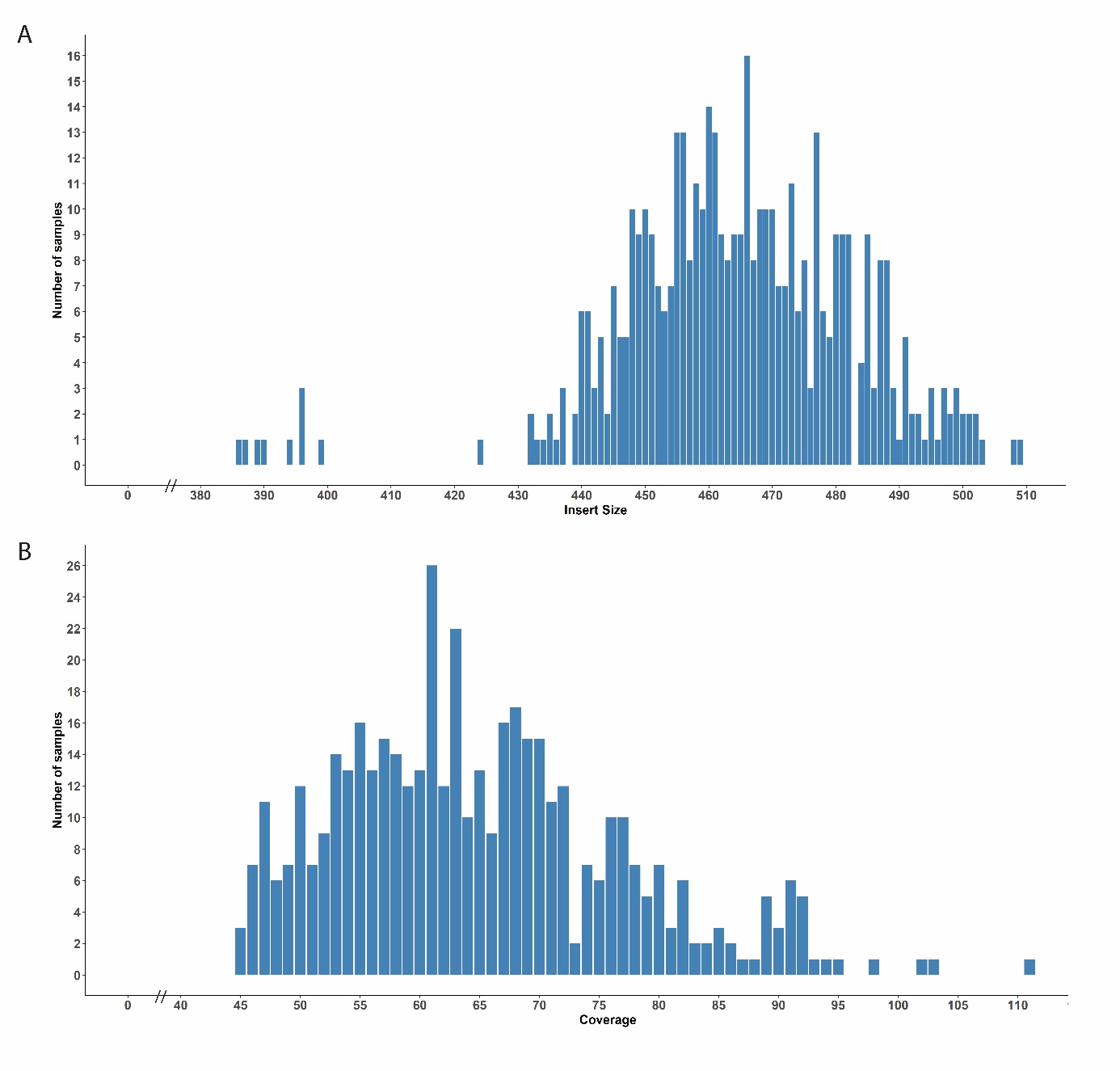


**Supplementary Figure 1: Distribution of insert size and coverage for genome sequencing of all 450 samples.**(A) Median insert size was 464 and interquartile range (IQR) was 454-477. (B) Median coverage was 63 and IQR was 56-71.

###
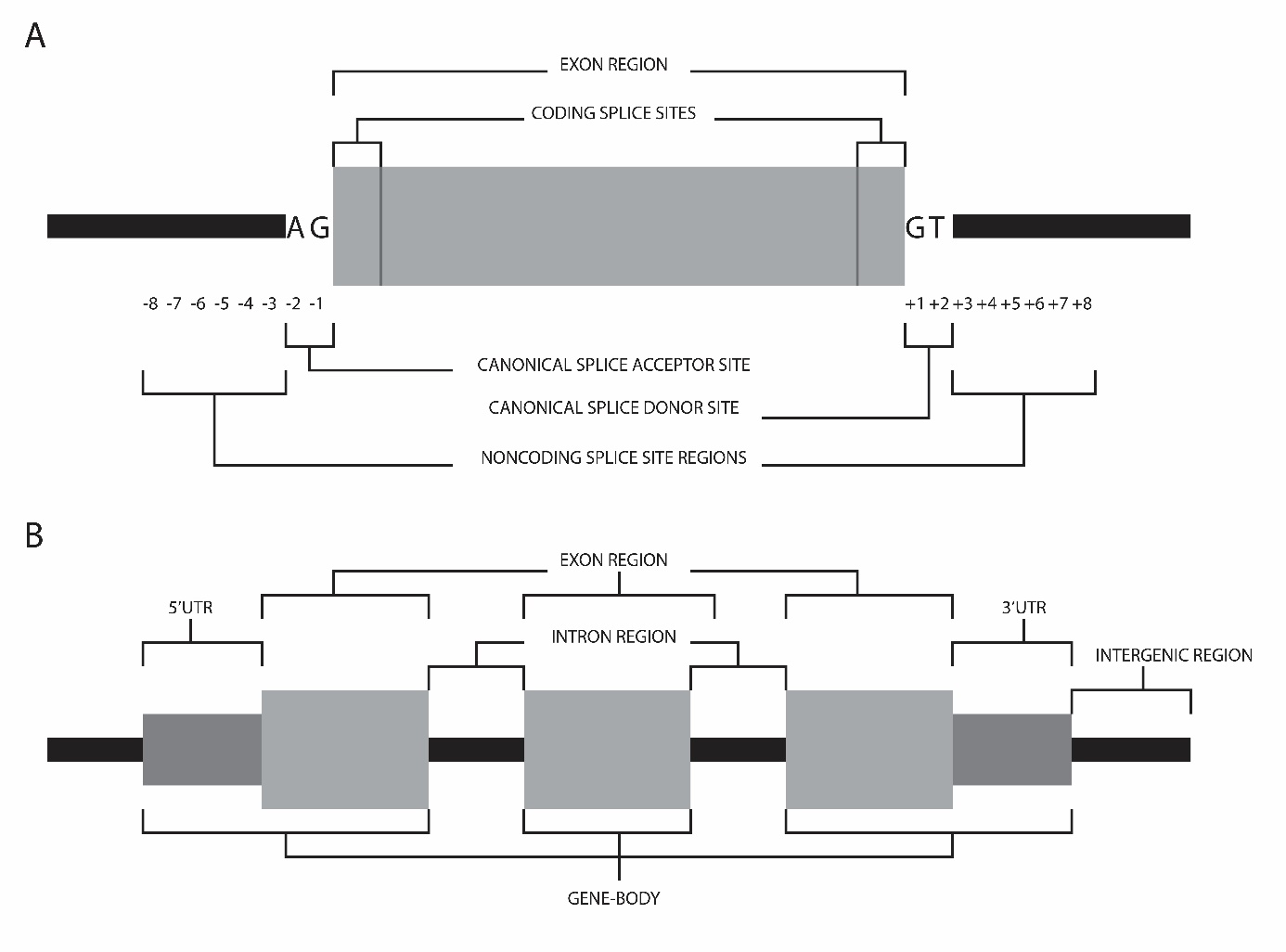


**Supplementary Figure 2: Graphic representation of ‘Exome-from-Genome’**For direct comparison of GS with the SOC pathway, only coding variants supplemented with SOC detectable NDD related pathogenic variants in the rest of the gene-body were analyzed. (A) To extract the coding variants from GS data, we defined the ‘exome from genome’ being all exons plus the first eight non-coding base pairs at both the 5’ and the 3’ sides of the exon (+/-8 bp). (B) The gene-body of NDD associated genes was defined as the sequence from the start of the 5’UTR till the end of the 3’UTR.


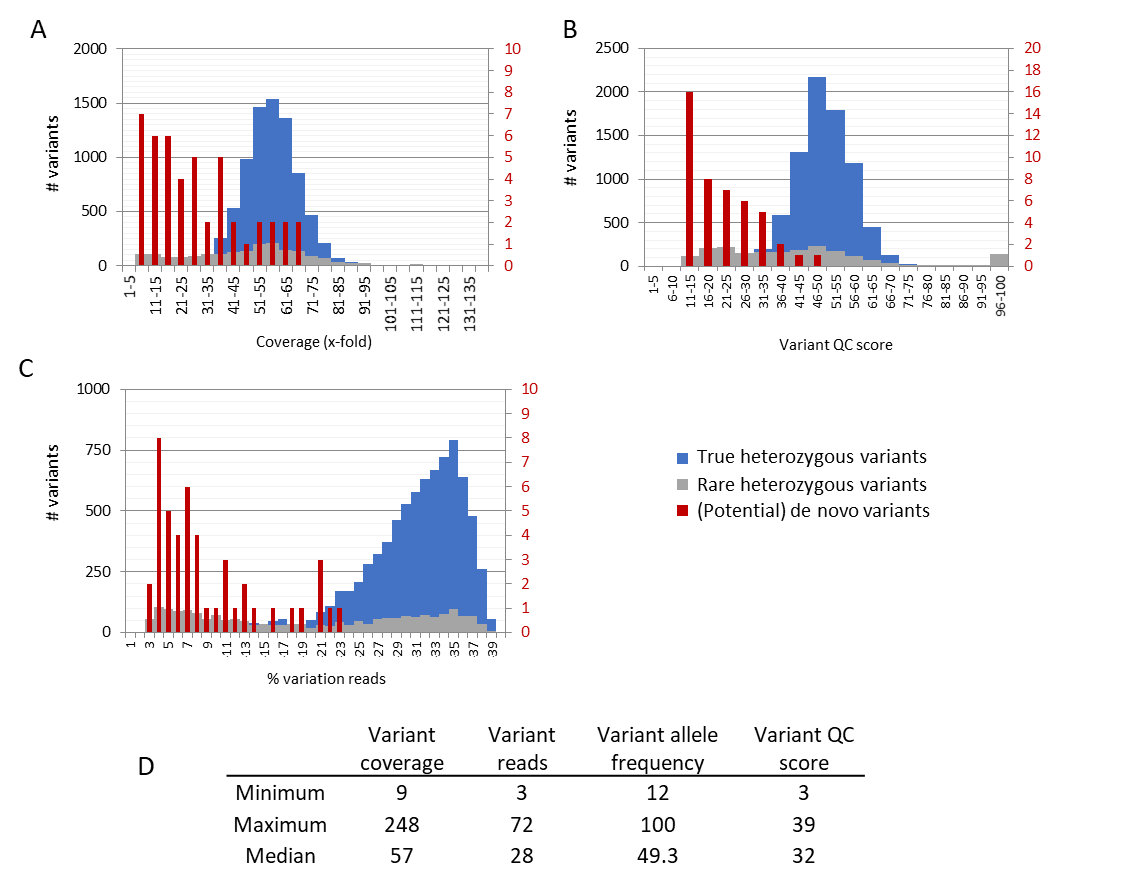


**Supplementary Figure 3: Distribution of quality metrics used for GS filtering**We first optimized our prioritization strategy based on Quality Control (QC) metrics, by analysing such metrics using a separate child-parent trio unrelated to this project and for which prior ES data was available. We first selected dominantly inherited variants in the child by taking informative homozygous coding SNVs identified the parents (e.g. transmitting one of these alleles to their offspring, resulting in heterozygosity in the child) with a GATK score>=500, at least 95% variant allele frequency, and known to gnomAD (e.g. common variant in population, and not platform specific technical artefact). This resulted in 8,073 variants, of which 7,970 (98.7%) also showed coverage in GS data. We next assessed the distribution on (A) coverage, (B) GATK variant quality score and (C) variant allele fraction for these positions in GS data in the child. (D) summarises the minimum, maximum and median values for coverage, reads, variation reads and GATK QC score.


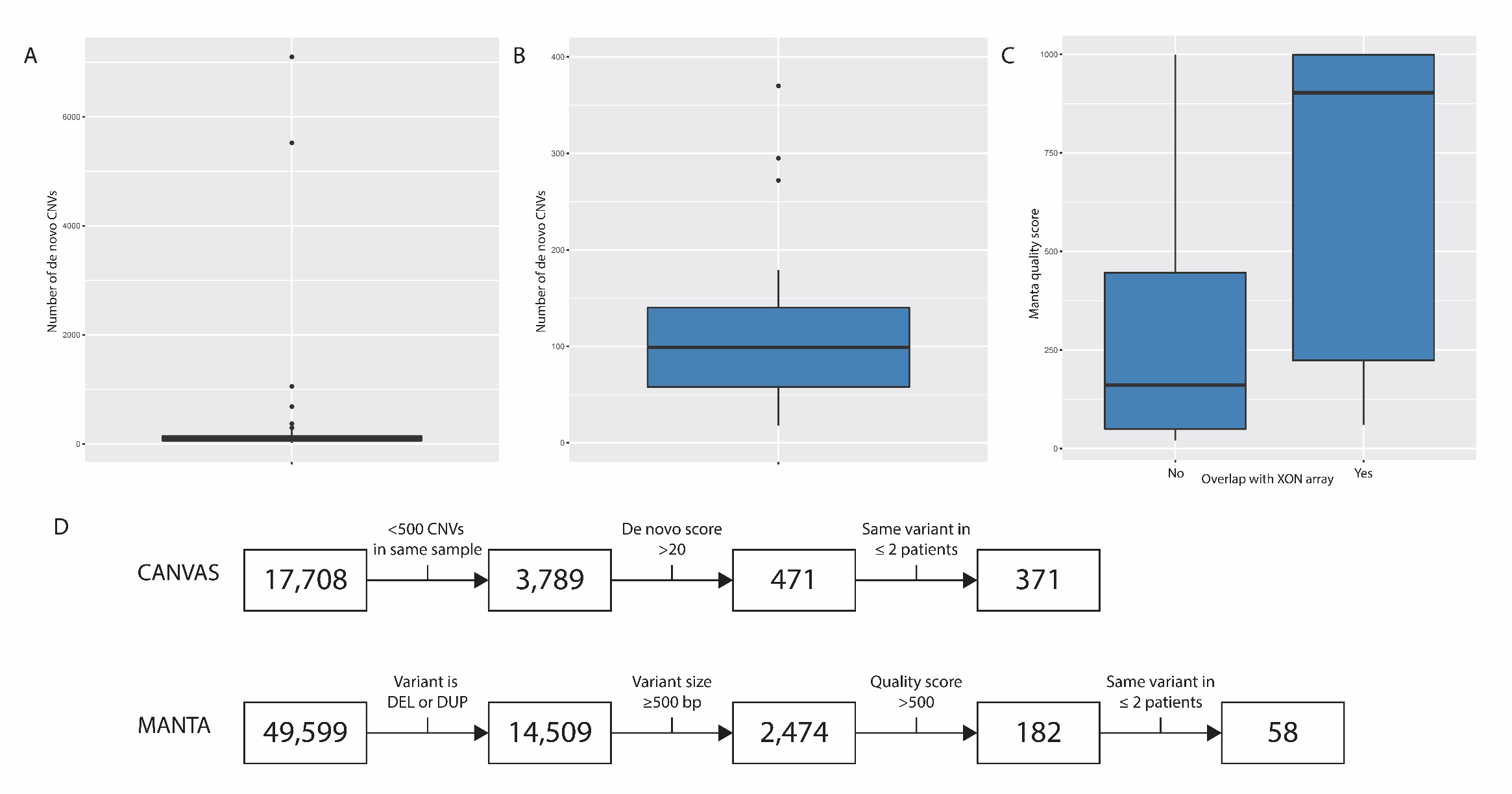


**Supplementary Figure 4: Distribution of quality metrics used for GS CNV filtering**We used the CNV and SV calling metrics of this cohort to establish a filter strategy. (A) Boxplot showing the number of *de novo* CANVAS CNV calls per sample. For quality control, outlier analysis was performed, from which we concluded a quality threshold of <500 de novo CNVs per sample for inclusion in subsequent analyses. For four families, ≥500 de novo CNVs were identified, leading to their exclusion. (B) Boxplot showing the number of *de novo* CANVAS CNV calls excluding the four samples with >500 CNVs. (C) Boxplot showing the overlap between the quality score of MANTA CNV calls and the XON array data from 29 samples. (D) We analysed the number of *de novo* variants that were detected by CANVAS. This resulted in 17,708 variants, of which 13,919 were attributable to the four indices with ≥500 *de novo* variants. We used the CANVAS de novo Phred-scaled quality score (dq20 flag) to omit false positive variant calls (n=3,318). We manually curated variants by excluding variants that occurred in >2 samples (n=100). From MANTA output (n=49,599), we only assessed the deletions and duplication that passed MANTA quality criteria (n=14,509), with a size equal or more than 500bp (n=2,474). We used XON array data from 29 samples to set a threshold at Q500, to omit possible false variant calls (n=2,292). Subsequently, we manually curated variants by excluding variants that occurred in >2 samples (n=124).


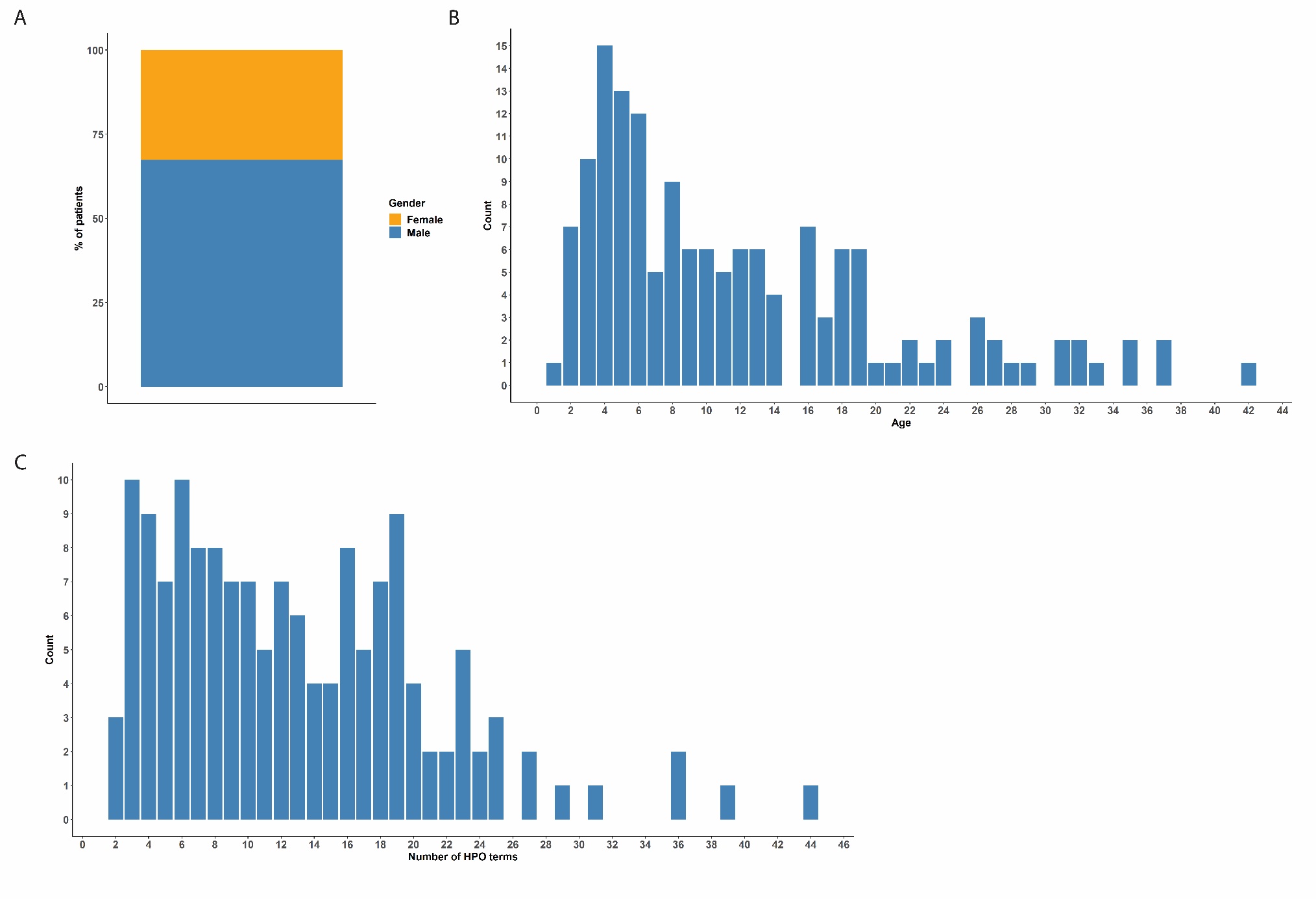


**Supplementary Figure 5: Demographic overview of cohort**Distribution of (A) males versus females in our cohort (B) age of the patient at time of inclusion (mean = 9 years and 6 months; IQR = 5 years and 3 months – 17 years and 7 months) and (C) the number of HPO terms per patient (median = 12 HPO terms; IQR = 6-18 HPO terms).

**Supplementary Tables**

**Supplementary Table 1: Patient and sequencing information**

*External File: Supplementary Table 1 - Patient + Sequencing details.xlsx*

**Supplementary Table 2: Thresholds for Qualimap assessment of GS data**

| **Statistics** | **Ideal** | **Warning** | **Error** |
| --- | --- | --- | --- |
| Insert size | ~450 bp | < 300 bp | <200 bp |
| GC content | ~41% | < 35% & > 50% | NA |
| Percentage duplicated reads | ~10% | > 20% | > 30% |
| Coverage | 50X | < 45X | < 20X |
| Percentage >20X coverage | ~90% | < 80% | < 70% |
| Error rate | ~0.6% | > 1% | >1.5% |
| Number of SNVs called | ~5,000,000 | < 4,250,000 | < 3,750,000 |

**Supplementary Table 3: Variant catalogue used for running ExpansionHunter on GS data**

| **Gene name** | **Genomic location (GRCh37)** | **Repeat unit** | **Reference repeat units** |
| --- | --- | --- | --- |
| *AFF2** | chrX:147582151-147582211 | GCC | 20 |
| *AR* | chrX:66765158-66765227 | GCA | 23 |
| *ARX** | chrX:25031779-25031808 | CGC | 9 |
| *ATN1* | chr12:7045879-7045936 | CAG | 19 |
| *ATXN1* | chr6:16327864-16327954 | TGC | 30 |
| *ATXN10* | chr22:46191234-46191304 | ATTCT | 14 |
| *ATXN2* | chr12:112036753-112036822 | GCT | 23 |
| *ATXN3* | chr14:92537353-92537386 | GCT | 11 |
| *ATXN7* | chr3:63898360-63898390 | GCA | 10 |
| *ATXN7* | chr3:63898390-63898402 | GCC | 4 |
| *ATXN8OS* | chr13:70713485-70713515 | CTA | 10 |
| *ATXN8OS* | chr13:70713515-70713560 | CTG | 15 |
| *C9orf72* | chr9:27573526-27573544 | GGCCCC | 3 |
| *CACNA1A* | chr19:13318672-13318711 | CTG | 13 |
| *CBL* | chr11:119076999-119077032 | CGG | 11 |
| *CNBP* | chr3:128891419-128891499 | CAGG | 20 |
| *CNBP* | chr3:128891499-128891539 | CAGA | 10 |
| *CNBP* | chr3:128891539-128891575 | CA | 18 |
| *COMP* | chr19:18896845-18896859 | GTC | 4 |
| *CSTB** | chr21:45196324-45196360 | CGCGGGGCGGGG | 3 |
| *DAB1* | chr1:57832716-57832790 | AAAAT | 14 |
| *DIP2B* | chr12:50898784-50898805 | GGC | 7 |
| *DMPK** | chr19:46273462-46273522 | CAG | 35 |
| *EIF4A3** | chr17:78120972-78120995 | CGTG | 5 |
| *FMR1** | chrX:146993568-146993628 | CGG | 45 |
| *FXN* | chr9:71652177-71652202 | A | 33 |
| *FXN* | chr9:71652202-71652220 | GAA | 6 |
| *HTT* | chr4:3076603-3076660 | CAG | 19 |
| *HTT* | chr4:3076666-3076693 | CCG | 9 |
| *JPH3* | chr16:87637893-87637935 | CTG | 14 |
| *NIPA1* | chr15:23086366-23086390 | CGC | 8 |
| *NOP56* | chr20:2633379-2633403 | GGCCTG | 4 |
| *NOP56* | chr20:2633403-2633421 | CGCCTG | 3 |
| *PABPN1* | chr14:23790681-23790699 | GCG | 6 |
| *PHOX2B* | chr4:41747989-41748049 | GCN | 20 |
| *PPP2R2B* | chr5:146258290-146258320 | GCT | 10 |
| *RFC1* | chr4:39350044-39350099 | AAGGG | 11 |
| *TBP* | chr6:170870994-170871105 | GCA | 37 |
| *TCF4* | chr18:53253386-53253458 | CAG | 24 |

(*) These alleles were extracted from the ExpansionHunter output and length of the expansion alleles were compared to reference values.

**Supplementary Table 4:** **Filters applied for the different types of variants and modes of inheritance**

| **Variant type** | **Inheritance** | **Filters** |
| --- | --- | --- |
| SNV | *De novo* | Exome from genome |
|  |  | Possible *de novo* |
|  |  | ≥ 5 variant reads |
|  |  | ≥ 20% variant allele frequency |
|  | Hemizygous | Exome from genome |
|  |  | Inheritance from mother |
|  |  | ≥ 5 variant reads |
|  |  | ≥ 80% variant allele frequency |
|  |  | ≤ 1% in-house frequency |
|  |  | Panel gene specific |
|  | Homozygous | Exome from genome |
|  |  | Inheritance from mother AND father |
|  |  | ≥ 5 variant reads |
|  |  | ≥ 80% variant allele frequency |
|  |  | ≤ 1% in-house frequency |
|  |  | Panel gene specific |
|  | Compound heterozygous | Exome from genome |
|  |  | Inheritance of one variant from mother and one different variant from father |
|  |  | ≥ 5 variant reads |
|  |  | ≥ 20% variant allele frequency |
|  |  | ≤ 1 in-house frequency |
|  |  | Panel gene specific |
| CNV | *De novo* | <500 CNVs |
|  |  | DENOVO_Q>20 |
|  |  | ≤ 2 samples |
|  | Hemizygous | <500 CNVs |
|  |  | Inheritance from mother |
|  |  | ≤ 2 samples |
| SV | *De novo* | Structural variant type = deletion or duplication |
|  |  | ≥ 500bp |
|  |  | Q > 500 |
|  |  | ≤ 2 samples |
|  | Hemizygous | Structural variant type = deletion or duplication |
|  |  | Inheritance from mother |
|  |  | ≥ 500bp |
|  |  | Q > 500 |
|  |  | ≤ 2 samples |
| STR | Anticipation | Gene = *AFF2*, *ARX*, *CSTB*, *DMPK*, *EIF4A3* or *FMR1* |
|  |  | Repeat length > pathogenic threshold^#^ |

# Pathogenic thresholds are in Supplementary Table 3

**Supplementary Table 5: Prioritization and interpretation criteria for the different variant types and modes of inheritance**

| **Variant type** | **Prioritization** | **Interpretation** | **Conclusion** |
| --- | --- | --- | --- |
| SNV | GnomAD ≤ 1% | 1. No pathogenic variant was detected that could explain the disease  2. VUS in disease-gene associated with (part of) phenotype  OR Pathogenic variant in candidate disease-gene with potential relationship to phenotype  3. Pathogenic variant in a disease-gene associated with phenotype | 1. No diagnosis  2. Possible diagnosis  3. Highly likely diagnosis |
|  | PhyloP ≥ 2.5 |  |  |
|  | CADD ≥ 20 |  |  |
|  | Alamut predicted effect* |  |  |
|  | SpliceAI prediction ≠ 0 \| 0 \| 0 \| 0 |  |  |
| CNV | Gene = NDD panel gene |  |  |
|  | Gene = other disease panel gene |  |  |
|  | Coding variant size ≥ 10kb |  |  |
| SV | Gene = NDD panel gene |  |  |
|  | Gene = other disease panel gene |  |  |
|  |  |  |  |
| STR | Repeat size > reference threshold |  |  |
|  |  |  |  |

* PolyPhen, SIFT and Align GVGD scores were used for missense predictions, whereas MutationTaster, SpliceSiteFinder, MaxEntScan, NNSPLICE, and GeneSplicer were used to predict possible splice effects.

**Supplementary Table 6: Human Phenotype Ontology terms for patient cohort (n=150)**

*External File: Supplementary Table 6 - HPO terms.xlsx*

**Supplementary Table 7: Representativeness of cohort**

|  | **This cohort  (n=150)** | |  | **Representative NDD cohort  (n=1,663)** | |  | **Adjusted p-value*** |
| --- | --- | --- | --- | --- | --- | --- | --- |
| **Intellectual disability** | **150** | **100%** |  | **1,663** | **100%** |  | **1.00** |
| IQ < 30 | 10 | 7% |  | 234 | 14% |  | 0.13 |
| IQ 30-50 | 7 | 5% |  | 134 | 8% |  | 1.00 |
| IQ 50-70 | 34 | 23% |  | 387 | 23% |  | 1.00 |
| *not specified* | *99* |  |  | *908* |  |  |  |
|  |  |  |  |  |  |  |  |
| **Epilepsy** | 15 | 10% |  | 350 | 21% |  | 0.01 |
|  |  |  |  |  |  |  |  |
| **Behavioral abnormality** | **103** | **69%** |  | **1,061** | **64%** |  | **1.00** |
| Autistic behavior | 59 | 39% |  | 547 | 33% |  | 1.00 |
| Schizophrenia | 1 | 1% |  | 5 | 0% |  | 1.00 |
| Sleep disturbance | 20 | 13% |  | 248 | 15% |  | 1.00 |
|  |  |  |  |  |  |  |  |
| **Macrocephaly** | **4** | **3%** |  | **100** | **6%** |  | **1.00** |
| **Microcephaly** | **8** | **5%** |  | **206** | **12%** |  | **0.12** |
| **Short stature** | **11** | **7%** |  | **244** | **15%** |  | **0.20** |
|  |  |  |  |  |  |  |  |
| **Abnormal morphology of the** |  |  |  |  |  |  |  |
| Brain | 20 | 13% |  | 346 | 21% |  | 0.50 |
| Heart | 6 | 4% |  | 78 | 5% |  | 1.00 |
| Genitourinary system | 18 | 12% |  | 289 | 17% |  | 1.00 |
| **Statistical analysis by two-tailed Fisher's exact, with p-values adjusted by Bonferroni correction for multiple testing* | | | | | | | |
|  | | |  |  |  |  |  |

**Supplementary Table 8: Overview of additional tests in standard of care pathway next to exome sequencing**

*External File: Supplementary Table 8 - overview SOC additional tests.xlsx*

**Supplementary Table 9: Overview of concordance between standard of care pathway versus genome sequencing pathway**

*External File: Supplementary Table 9 - overview SOC vs GS per patient.xlsx*

**Supplementary Table 10: Overview of concordant possible diagnoses between the standard of care and genome sequencing pathway**

| **Patient** | **Gender** | **Concordance** | **Gene** | **Genomic Mutation** | **cDNA** | **Protein** | **Inheritance** |
| --- | --- | --- | --- | --- | --- | --- | --- |
| 1 | male | yes | Multiple | (GRCh37)1q21.1q21.2(146493439-147389565)x3 | - | - | *de novo* |
| 6 | male | yes | *TBCD* | Chr17(GRCh37):g.80887150A>G | NC_000017.11(NM_005993.4):c.2852+3A>G | p.(?) | paternal |
|  |  |  |  | (GRCh37)17q25.3(80765557-81050791)x3 | - | - | maternal |
| 9 | male | yes | *TAF1* | ChrX(GRCh37):g.70587360G>C | NM_001286074.1:c.192G>C | p.(Lys64Asn) | hemizygous |
|  |  |  | *CUL4B* | ChrX(GRCh37):g.119679309G>A | NM_003588.3:c.964C>T | p.(Pro322Ser) | hemizygous |
|  |  |  | *IGF1R* | Chr15(GRCh37):g.99500344C>G | NM_000875.3:c.3777C>G | p.(Phe1259Leu) | *de novo* |
| 10 | male | yes | *SMARCA1* | ChrX(GRCh37):g.128640152G>A | NM_001282874.1:c.1003C>T | p.(Arg335Cys) | hemizygous |
| 13 | female | yes | *CUX2* | Chr12(GRCh37):g.111748343G>A | NM_015267.3:c.1757G>A | p.(Gly586Asp) | Paternal^#^ |
| 16 | male | yes | *USP9X* | ChrX(GRCh37):g.41007638C>G | NM_001039590.2:c.1436C>G | p.(Ala479Gly) | hemizygous |
| 19 | female | yes | *HGD* | Chr3(GRCh37):g.120363188C>T | NM_000187.3:c.752G>A | p.(Gly251Asp) | maternal |
|  |  |  |  | Chr3(GRCh37):g.120363283A>C | NM_000187.3:c.657T>G | p.(Asn219Lys) | paternal |
| 42 | female | yes | *CTCF* | Chr16(GRCh37):g.67645904C>G | NM_006565.3:c.832C>G | p.(Arg278Gly) | *de novo* |
| 43 | female | yes | *ZNF536* | (GRCh37)19q12(30875607-31056155)x3 | - | - | *de novo* |
| 45 | male | yes | *KDM3B* | Chr5(GRCh37):g.137722255C>T | NM_016604.3:c.1325C>T | p.(Ala442Val) | *de novo* |
| 50 | female | yes | *NF1* | Chr17(GRCh37):g.29528428G>T | NC_000017.11(NM_001042492.2):c.1186-1G>T | p.(?) | *de novo* |
| 52 | female | yes | *CPNE6* | Chr14(GRCh37):g.24546585C>T | NM_001280558.1:c.1687C>T | p.(Arg563*) | *de novo* |
| 54 | female | yes | *MYO9A* | Chr15(GRCh37):g.72338657A>G | NM_006901.3:c.248T>C | p.(Val83Ala) | maternal |
|  |  |  |  | Chr15(GRCh37):g.72170412T>C | NM_006901.3:c.5900A>G | p.(Glu1967Gly) | paternal |
| 58 | male | yes | *DNMT3A* | Chr2(GRCh37):g.25470497C>T | NM_022552.4:c.977G>A | p.(Arg326His) | *de novo* |
| 59 | male | yes | *ARID5A* | Chr2(GRCh37):g.97217495C>A | NM_001319085.1:c.1392C>A | p.(Tyr464*) | *de novo* |
| 60 | male | yes | *PCLO* | Chr7(GRCh37):g.82451924T>C | NM_033026.5:c.14678A>G | p.(His4893Arg) | paternal |
|  |  |  |  | Chr7(GRCh37):g.82544623T>C | NM_033026.5:c.12679A>G | p.(Ile4227Val) | maternal |
| 75 | male | yes | Multiple | (GRCh37)2q14.2q14.3(121995972-122727989)x1 | - | - | *de novo* |
| 78 | male | yes | *SMARCA1* | ChrX(GRCh37):g.128624163C>T | NM_001282874.1:c.1822G>A | p.(Ala608Thr) | hemizygous |
| 92 | male | yes | *LMBRD2* | Chr5(GRCh37):g.36117857_36117858del | NM_001007527.1:c.1284_1285del | p.(Tyr428*) | *de novo* |
| 101 | male | yes | *DDX3X* | ChrX(GRCh37):g.41205825T>C | NM_001356.4:c.1565T>C | p.(Ile522Thr) | hemizygous |
| 106 | male | yes | *ZNF536* | Chr19(GRCh37):g.30934971dup | NM_001352260.1:c.502dup | p.(Ala168Glyfs*78) | *de novo* |
| 108 | female | yes | *COL4A1* | Chr13(GRCh37):g.110825107T>A | NM_001845.4:c.3516A>T | p.(Gly1172=) | *de novo* |
| 111 | female | yes | *NTRK1* | Chr1(GRCh37):g.156849131A>G | NM_002529.3:c.2023A>G | p.(Ile675Val) | homozygous |
| 122 | male | yes | *PLCB1* | Chr20(GRCh37):g.8678396A>G | NM_015192.3:c.1133A>G | p.(His378Arg) | *de novo* |
| 129 | male | yes | *ATRX* | ChrX(GRCh37):g.76939664T>A | NM_000489.3:c.1084A>T | p.(Thr362Ser) | hemizygous |
| 130 | male | yes | *KIF4A* | ChrX(GRCh37):g.69615597A>G | NM_012310.4:c.2309A>G | p.(Asp770Gly) | hemizygous |
| 131 | female | yes | *KCTD13* | Chr16(GRCh37):g.29923369T>C | NM_178863.4:c.416A>G | p.(Gln139Arg) | *de novo* |
| 134 | male | yes | *GRM1* | Chr6(GRCh37):g.146755420_146755421delinsT | NM_001278064.1:c.3073_3074delinsT | p.(Pro1025Tyrfs*6) | *de novo* |
|  |  |  | *PLXNA3* | ChrX(GRCh37):g.153692310del | NM_017514.4:c.1564del | p.(Ala522Profs*15) | hemizygous |
| 136 | male | yes | *TCF20* | Chr22(GRCh37):g.42607236A>C | NM_001378418.1:c.4076T>G | p.(Ile1359Ser) | *de novo* |
|  |  |  | *OCRL* | ChrX(GRCh37):g.128692855C>G | NM_001318784.1:c.602C>G | p.(Pro201Arg) | hemizygous |
| 143 | male | yes | *IQSEC2* | ChrX(GRCh37):g.53277979G>A | NM_001111125.2:c.2383C>T | p.(Arg795Trp) | hemizygous |
| 150 | male | yes | *RIMS1* | (GRCh37)6q13(71825150-72807010)x1 | - | - | *de novo* |

*^#^ This variant was detected as mosaic variant in the father.*

**Supplementary Table 11: Possible diagnoses detected in the genome sequencing pathway only**

| **Patient** | **Gender** | **Concordance** | **Gene** | **Genomic Mutation** | **Size** | **Inheritance** |
| --- | --- | --- | --- | --- | --- | --- |
| 7 | male | GS+ | *CD226* | (GRCh37)18q22.2(67590464-67643093)x1 | 52 kb | *de novo* |
| 11 | male | GS+ | *ZNF536* | (GRCh37)19q12(30795567-30976083)x3 | 180 kb | *maternal^#^* |
| 83 | male | GS+ | *RERE* | (GRCh37)1p36.23(8780763-8825607)x1 | 44 kb | *de novo* |
| 148 | male | GS+ | Multiple | (GRCh37)Xp11.22q12(53953746-65852794)x2[0.3]* | 11.9 Mb | *de novo* |

*^#^ This variant was detected as mosaic variant in the mother.
*This mosaic pericentric gain is indicative for the presence of a supernumerary marker chromosome (SMC) in 30% of the cells.*
